# The first microbiological contamination assessment by deep-sea drilling and coring by the D/V *Chikyu* at the Iheya North hydrothermal field in the Mid-Okinawa Trough (IODP Expedition 331)

**DOI:** 10.3389/fmicb.2013.00327

**Published:** 2013-11-08

**Authors:** Katsunori Yanagawa, Takuro Nunoura, Sean M. McAllister, Miho Hirai, Anja Breuker, Leah Brandt, Christopher H. House, Craig L. Moyer, Jean-Louis Birrien, Kan Aoike, Michinari Sunamura, Tetsuro Urabe, Michael J. Mottl, Ken Takai

**Affiliations:** ^1^Subsurface Geobiology Advanced Research Project, Japan Agency for Marine-Earth Science and TechnologyYokosuka, Japan; ^2^Department of Earth and Planetary Science, University of TokyoTokyo, Japan; ^3^Department of Biology, Western Washington UniversityBellingham, WA, USA; ^4^Geomicrobiology, Federal Institute for Geosciences and Natural ResourcesHannover, Germany; ^5^Department of Geosciences, The Pennsylvania State University, University ParkPA, USA; ^6^Laboratoire Microbiologie Environnements Extremes, Institut Universitaire Européen de la MerPlouzane, France; ^7^Center for Deep Earth Exploration, Japan Agency for Marine-Earth Science and Technology, Yokohama Institute for Earth ScienceYokohama, Japan; ^8^Department of Oceanography, University of HawaiiHonolulu, HI, USA

**Keywords:** subseafloor biosphere, contamination, IODP Expedition, *Chikyu*, hydrothermal field

## Abstract

During the Integrated Ocean Drilling Program (IODP) Expedition 331 at the Iheya North hydrothermal system in the Mid-Okinawa Trough by the D/V *Chikyu*, we conducted microbiological contamination tests of the drilling and coring operations. The contamination from the drilling mud fluids was assessed using both perfluorocarbon tracers (PFT) and fluorescent microsphere beads. PFT infiltration was detected from the periphery of almost all whole round cores (WRCs). By contrast, fluorescent microspheres were not detected in hydrothermally active core samples, possibly due to thermal decomposition of the microspheres under high-temperature conditions. Microbial contamination from drilling mud fluids to the core interior subsamples was further characterized by molecular-based evaluation. The microbial 16S rRNA gene phylotype compositions in the drilling mud fluids were mainly composed of sequences of Beta- and Gammaproteobacteria, and Bacteroidetes and not archaeal sequences. The phylotypes that displayed more than 97% similarity to the sequences obtained from the drilling mud fluids were defined as possible contaminants in this study and were detected as minor components of the bacterial phylotype compositions in 13 of 37 core samples. The degree of microbiological contamination was consistent with that determined by the PFT and/or microsphere assessments. This study suggests a constructive approach for evaluation and eliminating microbial contamination during riser-less drilling and coring operations by the D/V *Chikyu*.

## Introduction

Scientific ocean drilling has demonstrated that microbial populations are ubiquitously detectable in deep marine subsurface environments, even at a depth of 1626 m below the seafloor (mbsf) (Roussel et al., 2008). Microbial cell abundance in subseafloor sediments is known to differ by approximately five orders of magnitude at different site locations, depths and geophysical-geochemical conditions, and the global subseafloor microbial cell abundance is estimated to be 2.9 × 10^29^ cells, thereby representing 0.6% of the total living biomass on Earth (Kallmeyer et al., 2012). To elucidate microbial diversity, functions, metabolic processes and geochemical interactions in subseafloor microbial ecosystem, core samples from scientific drilling expeditions are conventionally investigated via molecular biological analyses targeting 16S rRNA and functional genes, metabolic activity measurements and cultivation experiments (Fry et al., 2008; Orcutt et al., 2011). These attempts have revealed the presence of uncultivated, phylogenetically diverse and physiologically unknown microorganisms.

To better understand the indigenous subseafloor microbial populations and their functions, microbiologists must properly evaluate the potential microbial contamination from the drilling fluids into the core samples during the drilling and coring processes, particularly for core samples obtained from (extremely) low biomass environments. The contamination from the drilling fluids may also impact the interstitial water geochemistry, which is a source of important insights into the biogeochemical interactions and *in situ* functions of the subseafloor microbial communities. For scientific drilling expeditions that have dealt with the investigation of subseafloor microbial communities during the Ocean Drilling Program (ODP) and Integrated Ocean Drilling Program (IODP) using the D/V *JOIDES Resolution*, perfluorocarbon tracers (PFT) and particulate tracers (fluorescent microspheres) have been used to assess contamination from the drilling fluids (Smith et al., 2000; House et al., 2003; Lever et al., 2006). Contamination has often been detected in the core periphery and has occasionally been observed even in the interior parts.

The drilling facilities of the D/V *Chikyu*, a novel scientific drilling platform in the IODP, differ from those of the D/V *JOIDES Resolution*. The principal difference is the riser-drilling system of the D/V *Chikyu*, which requires a drilling mud fluid circulation device. The drilling mud fluid is composed of a mixture of freshwater and clay minerals or a mixture of surface seawater and organic compounds. Hence, the risk of contamination from the drilling mud fluids should be considered in the subsequent microbiological and geochemical studies. A previous study reported microbial growth in the circulation mud fluid of the riser-drilling operation by the D/V *Chikyu*, and 16S rRNA gene sequences of *Xanthomonas* were observed in the drilling mud fluids, which are composed of organic compounds (Masui et al., 2008). The D/V *Chikyu* can also operate riser-less drilling, for which surface seawater is often used, and occasionally drilling mud fluid is circulated to push out the core cuttings. However, the microbiological contamination of core samples obtained from the drilling and coring operations of the D/V *Chikyu* has not yet been evaluated.

The IODP Expedition 331 provided the first microbiology-dedicated opportunity to use the D/V *Chikyu* to study the subseafloor microbial communities associated with the hydrothermal system of the Iheya North field in the Mid-Okinawa Trough (Takai et al., 2011). During this expedition, one of the important scientific objectives was to clarify the possible boundary between the habitable and the uninhabitable zones of microbial life in the subseafloor hydrothermal environments. Hence, contamination assessment was a significant requirement to justify the possible existence of indigenous microbial populations and functions in such boundary habitats influenced by subseafloor hydrothermal fluid flows. We report here the first quantitative contamination tests of riser-less drilling cores using PFT and fluorescent microspheres during the IODP Expedition on the D/V *Chikyu*. Contamination from the drilling mud fluids into the microbiological subsamples was also evaluated by molecular biological analysis, in which the 16S rRNA gene sequences identified in the supplied drilling mud fluids were used as microbial molecular tracers to distinguish microbial contaminants from potential indigenous populations in the samples. This study shows the applicability of these contamination assessments for a variety of subseafloor environments and conditions sampled during the D/V *Chikyu* scientific expedition.

## Materials and methods

### Sampling sites, drilling operations, and sample collection

**Figure 1:**
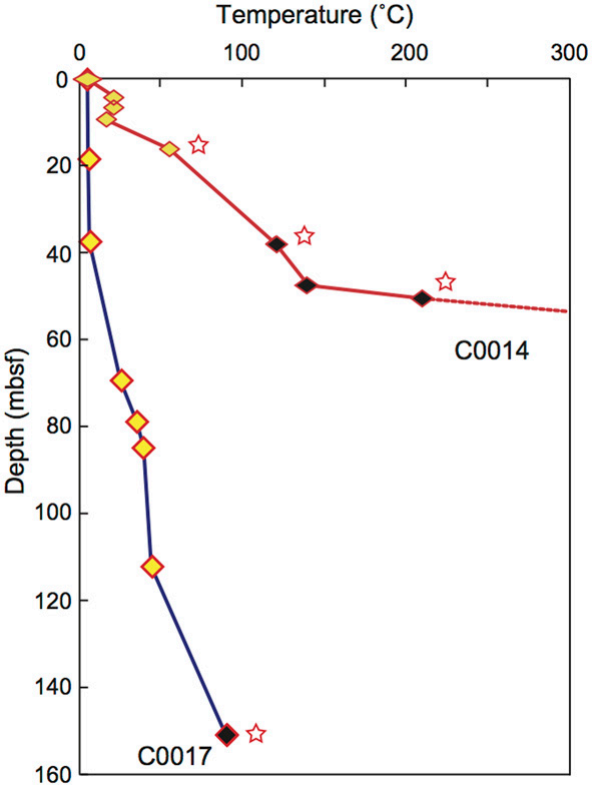
**Depth profile of temperature at Sites C0014 (red) and C0017 (blue), as measured by the by APCT-3 temperature shoe and thermoseal strip taped to the outer surface of the core liner**. Yellow, APCT-3; black, thermoseal strip. Stars; minimum values. Modified from Takai et al. (2011).

IODP Expedition 331 was conducted at the Iheya North hydrothermal field in the Mid-Okinawa Trough using the D/V *Chikyu* in September 2010. The study area is covered with thick terrigenous sediments, hemi-pelagic sediments and pumiceous deposits (Takai et al., 2011). We drilled five sites and collected core samples from the subseafloor environments beneath the hydrothermal field. IODP Site C0016 was located on the active hydrothermal mound of the North Big Chimney (NBC). The four drilling sites were located at 100 m (C0013), 450 m (C0014), and 1550 m (C0017) east and 600 m (C0015) northwest from the NBC. The hydraulic piston coring system (HPCS), the extended punch coring system (EPCS) and the extended shoe coring system (ESCS) were used for most of the coring in this expedition (See IODP Expedition 331 CDEX Technical Report; Center for Deep Earth Exploration, 2010) (Table 1). As drilling at Site C0016 yielded low recovery of cores, here, we describe microbiological contamination results for the other four sites. Core samples were retrieved at depths of up to 55 mbsf (C0013), 137 mbsf (C0014), and 151 mbsf (C0017). Most of the samples collected in this expedition were composed of pumiceous volcaniclastic gravels, breccias and interbedded hemipelagic mud (Takai et al., 2011). *In situ* temperatures at Sites C0013 and C0014 were measured using an advanced piston corer temperature tool (APCT-3) and thermoseal strips (Nichiyu Giken Co., Ltd., Kawagoe, Japan) at Site C0014 and C0017 (Figure 1) (Takai et al., 2011).

**Table 1:** **Results of contamination test using PFT, fluorescent microsphere and the 16S rRNA gene amplification**.

**Hole-section**	**Drilling operation**	**Depth (mbsf)**	**Core description**	**Microspheres/ml sediment**		**PFT (g/ml sediment)**		**PCR amplification of 16S rRNA genes**	**Contamination level^b^**
				**Interior**	**Exterior**	**Interior**	**Exterior**		
C0013B-1T-1	EPCS	0.6	Hydrothermal clay	N.D.^a^	5.8×10^2^				
C0013B-1T-1	EPCS	1.0	Hydrothermal gravel, clast-supported	N.D.	4.2×10^2^			+	0%
C0013D-1H-1	HPCS	3.1	Sulfidic sand	1.7×10^4^	6.6×10^4^			+	52%
C0013D-1H-2	HPCS	4.3	Hydrothermal grit, clast-supported	5.8×10^2^	8.4×10^4^				
C0013D-1H-4	HPCS	6.9	Hydrothermal gravel, matrix-supported	N.D.	1.2×10^4^				
C0013D-1H-5	HPCS	7.7	Hydrothermal gravel, matrix-supported	N.D.	N.D.				
C0013E-1H-2	HPCS	0.2	Sulfidic sand	N.D.	3.9×10^2^				
C0013E-1H-2	HPCS	1.1	Sulfidic sand	N.D.	N.D.				
C0013E-1H-4	HPCS	2.4	Sulfidic sand	N.D.	N.D.				
C0013E-1H-5	HPCS	4.3	Hydrothermal clay	N.D.	N.D.				
C0013E-1H-6	HPCS	5.4	Hydrothermal grit, matrix-supported	N.D.					
C0013E-5H-1	HPCS	17.0	Hydrothermal clay	N.D.					
C0013F-1H-2	HPCS	0.2	Sulfidic sand	N.D.	N.D.	4.3×10^−6^	3.3×10^−3^		
C0013F-1H-3	HPCS	1.4	Hydrothermal clay	N.D.	1.5×10^2^				
C0013F-1H-5	HPCS	3.1	Hydrothermal clay	N.D.	N.D.	N.D.	1.0×10^−5^		
C0014B-1H-1	HPCS	0.3	Clay	N.D.	N.D.			+	0%
C0014B-1H-1	HPCS	1.1	Clay	N.D.	N.D.	1.0×10^−5^	5.3×10^−5^	+	0%
C0014B-1H-2	HPCS	2.4	Clay	N.D.	N.D.	N.D.	1.3×10^−5^	+	0%
C0014B-2H-3	HPCS	8.5	Pumiceous grit, clast-supported	N.D.	3.6×10^4^	N.D.	5.3×10^−5^	+	0%
C0014B-2H-7	HPCS	12.2	Hydrothermal clay	1.8×10^2^	N.D.			+	0%
C0014B-2H-10	HPCS	14.3	Hydrothermal clay	N.D.	N.D.	N.D.	8.2×10^−6^	+	0%
C0014B-3H-2	HPCS	17.2	Hydrothermal clay	N.D.	2.2×10^5^				
C0014B-3H-5	HPCS	19.2	Hydrothermal clay	N.D.	6.3×10^3^	1.3×10^−6^	7.8×10^−5^		
C0014B-3H-9	HPCS	23.2	Hydrothermal clay	N.D.	N.D.	8.0×10^−7^	9.9×10^−6^		
C0014B-4H-2	HPCS	26.2	Hydrothermal clay	N.D.	5.8×10^2^				
C0014B-4H-3	HPCS	27.5	Hydrothermal gravel, matrix-supported	N.D.	N.D.	N.D.	3.9×10^−6^		
C0014B-4H-4	HPCS	28.8	Hydrothermal gravel, matrix-supported	N.D.	N.D.				
C0014B-4H-6	HPCS	31.1	Hydrothermal clay	N.D.	N.D.				
C0014B-4H-7	HPCS	32.6	Hydrothermal clay	N.D.	N.D.				
C0014B-4H-8	HPCS	33.7	Hydrothermal clay	N.D.	N.D.	N.D.	N.D.		
C0014B-5H-12	HPCS	40.6	Hydrothermal clay	N.D.	N.D.	2.6×10^−5^	6.0×10^−4^		
C0014B-5H-14	HPCS	42.3	Hydrothermal gravel, matrix-supported	N.D.	N.D.				
C0014B-5H-15	HPCS	43.8	Hydrothermal gravel, matrix-supported	N.D.	N.D.	N.D.	1.5×10^−3^		
C0014D-1H-1	HPCS	0.2	Clay	N.D.	N.D.	N.D.	N.D.	+	0%
C0014D-1H-2	HPCS	2.0	Sandy silt	N.D.	3.6×10^2^			+	0%
C0014D-1H-3	HPCS	3.1	Pumiceous gravel, matrix-supported	N.D.	N.D.	2.0×10^−5^	1.9×10^−6^	+	0%
C0014D-1H-4	HPCS	4.2	Pumiceous grit, clast-supported	N.D.	5.5×10^2^	1.3×10^−5^	2.1×10^−4^	+	1%
C0014D-2H-1	HPCS	6.7	Pumiceous gravel, clast-supported	1.4×10^4^	7.7×10^3^	1.0×10^−4^	1.1×10^−4^	+	10%
C0014D-2H-2	HPCS	8.6	Pumiceous gravel, clast-supported	N.D.	N.D.	2.6×10^−6^	1.7×10^−5^	+	0%
C0014D-2H-3	HPCS	10.2	Gradation from clayey hydrothermal sand to pumiceous grit, matrix-supported	N.D.	3.9×10^2^			+	0%
C0014D-2H-4	HPCS	11.4	Hydrothermal clay	N.D.	1.5×10^2^	N.D.	1.7×10^−6^	+	0%
C0014D-2H-6	HPCS	12.8	Hydrothermal clay	N.D.	1.2×10^3^	N.D.	1.7×10^−6^		
C0014E−1H-3	HPCS	18.5	Hydrothermal clay	N.D.	9.4×10^3^				
C0014E−1H-4	HPCS	19.7	Hydrothermal clay	N.D.	3.1×10^3^	N.D.	5.8×10^−6^		
C0014E−1H-5	HPCS	20.7	Hydrothermal clay	N.D.	N.D.				
C0014E−1H-6	HPCS	22.1	Hydrothermal clay	N.D.		N.D.	N.D.		
C0014E−2H-4	HPCS	27.4	Hydrothermal clay	N.D.	N.D.				
C0014E−2H-5	HPCS	29.1	Hydrothermal clay	N.D.	N.D.				
C0014E−2H-6	HPCS	30.8	Hydrothermal gravel, matrix-supported	N.D.	N.D.	N.D.	2.5×10^−5^		
C0014E−2H-7	HPCS	32.0	Hydrothermal clay	N.D.	N.D.				
C0014E−2H-8	HPCS	33.3	Hydrothermal clay	N.D.	N.D.	N.D.	5.6×10^−5^		
C0014G-1H-1	HPCS	0.3	Clay	N.D.	N.D.	9.6×10^−7^	9.1×10^−4^	+	5%
C0014G-1H-2	HPCS	1.8	Silty clay	N.D.	N.D.			+	1%
C0014G-1H-3	HPCS	3.7	Sandy clay	1.9×10^2^	2.5×10^3^			+	4%
C0014G-1H-4	HPCS	4.1	Pumiceous gravel, matrix-supported	N.D.	N.D.	1.5×10^−6^	2.3×10^−4^	+	10%
C0014G-1H-5	HPCS	5.9	Hydrothermal clay	N.D.	N.D.			+	5%
C0014G-1H-6	HPCS	7.8	Pumiceous gravel, clast-supported	2.9×10^2^	N.D.			+	26%
C0014G-2H-5	HPCS	15.8	Hydrothermal clay	N.D.	1.5×10^2^	N.D.	N.D.	+	2%
C0014G-2H-7	HPCS	17.6	Hydrothermal clay	N.D.	4.4×10^2^				
C0014G-3H-5	HPCS	22.7	Hydrothermal clay	N.D.	N.D.	N.D.			
C0014G-3H-8	HPCS	25.3	Hydrothermal clay	N.D.	N.D.				
C0014G-4H-2	HPCS	29.2	Hydrothermal clay	N.D.	N.D.	N.D.	N.D.		
C0014G-4H-5	HPCS	31.0	Hydrothermal clay	N.D.	N.D.				
C0014G-4H-7	HPCS	32.6	Hydrothermal clay	N.D.	N.D.				
C0014G-4H-9	HPCS	34.6	Hydrothermal gravel, matrix-supported	4.0×10^3^	3.3×10^3^				
C0014G-4H-10	HPCS	35.6	Hydrothermal clay	N.D.	N.D.				
C0014G-4H-11	HPCS	37.1	Hydrothermal clay	N.D.	1.5×10^3^				
C0014G-5H-3	HPCS	38.1	Hydrothermal clay	N.D.	N.D.	N.D.	1.4×10^−4^		
C0014G-6H-2	HPCS	47.6	Hydrothermal clay	N.D.	N.D.				
C0014G-6H-3	HPCS	47.8	Hydrothermal clay	N.D.	N.D.	5.8×10^−7^	2.8×10^−6^		
C0014G-9X-2	ESCS	55.7	Hydrothermal gravel, matrix-supported			3.0×10^−5^	3.9×10^−4^		
C0014G-12H-3	HPCS	65.4	Hydrothermal clay	N.D.	N.D.	N.D.	1.2×10^−6^		
C0014G-13T-1	EPCS	67.5	Hydrothermal clay			N.D.			
C0014G-14T-2	EPCS	71.7	Hydrothermal clay			1.0×10^−6^	1.3×10^−4^		
C0014G-16T-1	EPCS	76.4	Hydrothermal clay			1.3×10^−5^	4.3×10^−6^		
C0014G-17T-2	EPCS	81.2	Hydrothermal clay			8.2×10^−7^	8.3×10^−6^		
C0015B-1H-1	HPCS	0.3	Pumiceous gravel, matrix-supported	N.D.	N.D.	N.D.	1.3×10^−6^		
C0015B-1H-3	HPCS	3.4	Mud-supported bioclastic gravel	N.D.	4.4×10^2^	N.D.	3.6×10^−6^		
C0015B-1H-5	HPCS	5.6	Clay	N.D.	1.8×10^2^				
C0015C-1H-1	HPCS	6.9	Sand	4.4×10^4^	1.5×10^4^				
C0015C-1H-3	HPCS	8.8	Sand	N.D.	1.5×10^2^	1.3×10^−5^	1.7×10^−5^		
C0017A-1H-1	HPCS	0.7	Clay	N.D.	1.3×10^3^	N.D.	3.4×10^−5^	+	0%
C0017A-1H-5	HPCS	6.4	Gradation from silty clay to calcareous sand	N.D.	2.9×10^3^	N.D.	1.1×10^−4^	+	0%
C0017B-1H-2	HPCS	10.8	Clay	N.D.	2.5×10^4^	N.D.	1.9×10^−4^	+	0%
C0017B-1H-5	HPCS	14.8	Clay	N.D.	2.3×10^2^	N.D.	7.4×10^−5^	+	0%
C0017C-1H-2	HPCS	20.1	Pumiceous gravel, matrix-supported	2.9×10^2^	7.0×10^4^	N.D.	5.1×10^−5^	+	0%
C0017C-1H-5	HPCS	24.6	Sandy clay	N.D.	3.1×10^3^				
C0017C-1H-7	HPCS	26.6	Pumiceous gravel, clast-supported	N.D.	0.0×10^0^	N.D.	2.8×10^−6^	+	1%
C0017C-2H-1	HPCS	28.4	Pumiceous gravel, clast-supported	1.9×10^4^	9.6×10^3^			+	5%
C0017C-2H-2	HPCS	30.0	Sandy clay	N.D.	1.5×10^2^	N.D.	7.2×10^−6^	+	0%
C0017D-1H-3	HPCS	63.6	Pumiceous gravel, matrix-supported	N.D.	N.D.	4.0×10^−6^	1.5×10^−5^	+	0%
C0017D-1H-5	HPCS	66.4	Pumiceous gravel, clast-supported	N.D.	N.D.	4.4×10^−6^	1.8×10^−6^		
C0017D-1H-6	HPCS	68.1	Volcaniclastic sand/sandstone	N.D.	N.D.	N.D.	1.4×10^−5^	+	0%
C0017D-2H-5	HPCS	74.9	Volcaniclastic mud/mudstone	N.D.	5.1×10^3^	N.D.	3.6×10^−6^	+	1%
C0017D-6X-1	ESCS	95.0	Clay			N.D.	2.9×10^−6^	+	0%
C0017D-7H-4	HPCS	108.2	Silty clay	N.D.	7.0×10^3^	2.2×10^−6^	1.9×10^−6^	+	0%
C0017D-9X-8	ESCS	130.1	Clay			2.6×10^−6^	3.8×10^−6^	+	0%
C0017D-10X-4	ESCS	136.1	Clay			1.2×10^−6^			
C0017D-11X-1	ESCS	141.1	Silty clay			6.6×10^−6^	7.4×10^−4^	+	55%

a N.D., not detected.

b Contamination level was estimated from proportion of potential contaminant sequences from drilling fluids in the microbial community.

Guar gum mud containing surface seawater and 0.8% (wt/v) guar gum or seawater gel mud containing freshwater (distilled seawater and/or chlorinated tap water), 5% bentonite, 0.1% sodium hydroxide, 0.1% lime and barite was used as the drilling fluid mud during IODP Expedition 331. The specific gravity of the seawater gel mud was controlled by the barite content. During the drilling operations at Sites C0013, C0014, and C0016, 10 ml samples of drilling mud fluids were arbitrarily collected from the storage tank onboard and stored at −80°C until onshore laboratory analysis. The retrieved cores were cut into 1.5-m-long whole round sections on deck, and whole round cores (WRCs) for microbiological study (~10–20 cm in length) were subsampled from the short sections of the cores. The subsamples for PFT and microsphere tests were obtained from the innermost parts (within 2 cm in radius) and the outer edges of the WRCs and placed in glass vials with tight butyl rubber stoppers or plastic tubes, respectively. The microbiological samples were obtained from the inner parts of the WRCs with a sterilized spatula and immediately stored at −80°C in heat-sealed laminated foil bags containing an oxygen scavenger.

### PFT test

Perfluoromethylcyclohexane (C_7_F_14_) was used as a chemical indicator of drilling fluid contamination. When the drill mud components of clay minerals and/or organic compounds were dissolved in surface seawater, PFT was adjusted to 1 ppm in the storage tank for the drilling mud fluids. The mud samples were occasionally sampled to measure the PFT concentrations in the drilling mud fluids and were stored in glass vials with tight butyl rubber stoppers. The PFT concentrations in the cored sediments and mud samples were measured following standard IODP procedures (Smith et al., 2000) with an Agilent Technologies model 6890N gas chromatograph (GC) with an electron capture detector (ECD) (Agilent Technologies, Santa Clara, CA). The GC was equipped with an HP-PLOT Al_2_O_3_ “M” deactivation column (30 m length; 0.53 mm interior diameter; 15 μm coating thickness). Helium and nitrogen were used as the carrier gas and make-up gas, respectively.

### Microsphere test

A suspension of 0.5-μm-sized fluorescent beads (Polysciences Inc., Warrington, Pa) was released in the core cutting shoe at the critical moment of impact against the seafloor and the targeted layers (Smith et al., 2000). The microsphere test was applied in some of the HPCS operations but not for the ESCS operation. The infiltration of microspheres was monitored according to the standard IODP procedures, as described previously (Smith et al., 2000).

### DNA extraction and prokaryotic 16s rRNA gene phylogenetic analysis

DNA was extracted from ~2 g of frozen innermost parts of WRCs or drilling mud fluids using the PowerMAX Soil DNA Isolation Kit (MO BIO Laboratories, Carlsbad, CA) according to the manufacturer's protocol, with slight modifications. Before cell disruption, the samples were incubated at 65°C for 5 min. The samples were then subjected to mechanical shaking for 10 min with a ShakeMaster (BioMedical Science, Tokyo, Japan). A blank DNA extraction was performed simultaneously as a negative control experiment during the extraction process.

The 16S rRNA gene fragments were amplified by PCR using the universal primer set Uni530F-907R (Nunoura et al., 2012) and an Archaea-specific primer set composed of Arch_530F, Arch2_530F, Nano_530F (Nunoura et al., 2012) and Arc958R (Delong, 1992). PCR amplification with LA Taq polymerase (TaKaRa Bio Inc., Otsu, Japan) was performed using a Veriti 96-well thermal cycler (Applied Biosystems, Foster City, CA). The amplification conditions were as follows: 40 cycles of denaturation at 96°C for 25 s, annealing at 50°C for 45 s, and extension at 72°C for 60 s for DNA fragments encoding 16S rRNA genes. PCR amplification of an extraction blank was used to assess experimental contamination. Cloning and sequencing of the PCR products were performed as described in Igisu et al. (2012). The Mothur Utility package was used for statistical analyses of the 16S rRNA sequence data (Schloss et al., 2009). Partial 16S rRNA gene sequences with more than 97% similarity were assigned to the same operational taxonomic unit (OTU). Representative sequences were aligned to the SILVA Reference Alignment using the NAST algorithm (Pruesse et al., 2007), followed by manual alignment. Phylogenetic affiliations were assigned by the maximum parsimony method with the SILVA SSU Ref111 Database in ARB software (Ludwig et al., 2004). The 16S rRNA gene sequences in the subseafloor core samples that displayed greater than 97% similarity with those of the drilling fluids were defined as potential contaminants.

### Nucleotide sequence accession numbers

The 16S rRNA gene sequences determined in this study have been deposited in the DDBJ/EMBL/GenBank databases under accession numbers AB824899 through AB825952.

## Results and discussion

### Contamination assessment under high-temperature condition

In the contamination test using fluorescent microspheres of core samples obtained from high-temperature regions of the subseafloor environments at IODP Sites C0013 and C0014, no microspheres or only very few microspheres were observed, even in the most outer surface of the WRCs (Table 1). *In situ* temperature was not measured at Site C0013, but most of the plastic core liners were melted by exposure to high temperatures. The core liners were made of butyrate plastic, the melting point of which ranges from 70 to 80°C. At Site C0014, a temperature gradient of 3–4°C/m was estimated by the APCT-3 and thermoseal strips (Figure 1) (Takai et al., 2011), and the microspheres were detected only in the WRCs at shallower depths. Because the fluorescent microsphere were latex beads formed from an amorphous polymer (polystyrene), most of the microspheres would be thermally degraded under high-temperature conditions, i.e., a glass transition temperature of ~95°C and melting temperature of ~240°C. By contrast, at Sites C0015 and C0017, where signatures of hydrothermal fluid inputs were not observed in the interstitial water geochemistry and APCT-3 temperature measurements, contamination assessment using the fluorescent microspheres could be completed (Table 1). Microspheres were not detected in the exterior part of 4 samples of a total of 19 samples at these 2 sites. This outcome suggests that the microspheres were not uniformly delivered to the exterior part of the core, in agreement with previous reports (Smith et al., 2000; House et al., 2003). The interior parts of the WRCs, which were used for microbiological studies, generally contained few fluorescent microspheres. However, microspheres were observed in only 10 samples, and a striking penetration of more than 10^4^ beads/ml sediment into the core interiors were observed in 4 samples; C0013D-1H-1, C0014D-2H-1, C0015C-1H-1, and C0017C-2H-1.

In contrast to the microsphere test, PFTs were detectable in the exterior parts of many WRCs, even those from the deepest part of the drilling holes (Table 1). The PFTs are a highly volatile material with an atmospheric boiling point of 76°C and thus should be present in the gas phase under the high-temperature conditions in the deeper sections of Sites C0013 and C0014. However, our results suggest that PFTs could be applicable for contamination monitoring even in the deep subsurface beneath the deep-sea hydrothermal field, presumably due to the cooling effect of the injection of drilling mud fluids and the *in situ* high hydrostatic pressures. Significant amounts of PFTs were detected in C0014B-1H-1, C0014B-5H-12, C0014D-1H3, C0014D-1H-4, C0014D-2H-1, C0014G-9X-2, C0014G-16T-1, and C0015C-1H-3 (Table 1).

### Drilling mud fluid contamination by different coring systems

The extent of the drilling mud fluid contamination was likely varied among the coring systems adopted: HPCS, EPCS and ESCS. Generally, the HPCS-recovered cores were less contaminated than the ESCS- and EPCS-recovered cores because the cored sediment was undisturbed in the HPCS-recovered cores. PFTs were detected in the interior of 15 of 44 WRCs (34%) obtained by the HPCS operation (Table 1). By contrast, we observed a significant concentration of PFTs in the interior of most of the WRCs obtained by ESCS or EPCS (7 of 9 cores; 78%). The result seemed to depend on the lithological properties of the core samples because the lithological transition of the subseafloor environment indeed impeded the HPCS operation during the expedition. HPCS could retrieve less-contaminated WRCs from several specific layers, such as the coarse-grained pumiceous gravel and the breccia localized in Site C0017 (depth of 26.6 and 30.0 mbsf). Such highly permeable layers tend to be highly influenced by the drilling fluid contamination but are expected to serve as the potential fluid flows and harbor larger populations of functional microbial communities in the subseafloor environment beneath the hydrothermal field. Consequently, HPCS yielded the lowest contamination and the best samples for subsequent study at any of the depths and lithological layers during the expedition by the D/V *Chikyu*.

### Microbial 16s rRNA gene phylotype compositions in the drilling fluids

**Figure 2:**
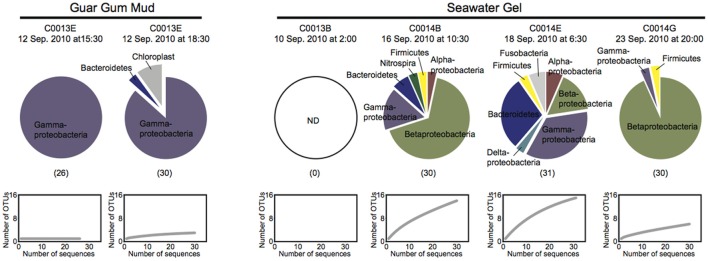
**Microbial 16S rRNA gene phylotype compositions and rarefaction curves for the clone libraries for the guar gum mud and the seawater gel mud, which were used as drilling mud fluids during the coring at Sites C0013 and C0014**. The 16S rRNA gene fragments were amplified with universal primer set 530F-907R. The numbers in parentheses indicate the number of clones.

The 16S rRNA gene sequences in the drilling fluids used for the drilling and coring operations at Sites C0013 and C0014 were analyzed to clarify the possible microbial sources of drilling fluid contamination (Figure 2). All of the 16S rRNA gene sequences obtained with the universal primer set were affiliated with bacterial phyla/divisions, and we did not detect any archaeal sequences, even when using the Archaea-specific primer set. The bacterial phylotype compositions in the drilling fluids composed of organic compounds (guar gum mud) were less diverse and were distinct from those in the drilling fluids composed of clay minerals (seawater gel) (Figure 2). Members of Vibrionaceae of Gammaproteobacteria comprised ~87% of total bacterial clone sequences in the guar gum mud, whereas the seawater gel drilling fluids displayed more diverse phylotype compositions, including Beta- and Gammaproteobacteria and Bacteroidetes. The sequences assigned to Betaproteobacteria included the orders Burkholderiales and Rhodocyclales. These orders have been identified among contamination-related sequences in previous studies but were not regarded as contaminants (Masui et al., 2008; Santelli et al., 2010). The Gammaproteobacteria sequences belonged to Alteromonadales, Oceanospirillales, Pseudomonadales, Vibrionales, and Xanthomonadales. All 16S rRNA gene sequences in these drilling fluids were derived from possibly mesophilic and heterotrophic bacterial components. No detectable 16S rRNA gene sequences were obtained from very freshly prepared drilling fluid of the seawater gel for the operation at Site C0013B (Figure 2). The seawater gel mud was likely semi-sterile because it was prepared with distilled seawater and/or chlorinated tap water. Thus, the diverse phylotypes observed in most of the seawater gel samples would be derived from the microbial populations that grew in the seawater gel mud fluids after preparation. *Xanthomonas* was previously reported as the predominant 16S rRNA gene sequence in the precirculation drilling mud fluids prepared for the riser-drilling in the *D/V Chikyu* shakedown cruise (Masui et al., 2008). The *Xanthomonas* DNA originally contaminated the xanthan gum reagent powder, which was added to the mud fluids to maintain the viscosity for the riser-drilling. However, xanthan gum was not used to prepare the mud fluids during IODP Expedition 331. Accordingly, our results revealed that *Xanthomonas* relatives accounted for only 3% of the 16S rRNA gene composition of seawater gel mud during the drilling at C0014B. The scientific drilling and coring operations by the *D/V Chikyu* adopt a variety of drilling fluid formulas for different expeditions, sites, holes and even during the operation of the same hole. Thus, the microbial populations in the drilling fluids and, consequently, the possible sources of microbial contamination of the core samples may vary in different drilling operation (riser or riser-less) and drilling fluids and at different times of preparation of drilling fluids. Periodic and repeated sampling of the drilling fluids as reference is therefore essential for microbiological investigations using core samples obtained from the *D/V Chikyu*.

### Comparison of microbial 16s rrna gene phylotype compositions in between drilling fluids and core samples

**Figure 3:**
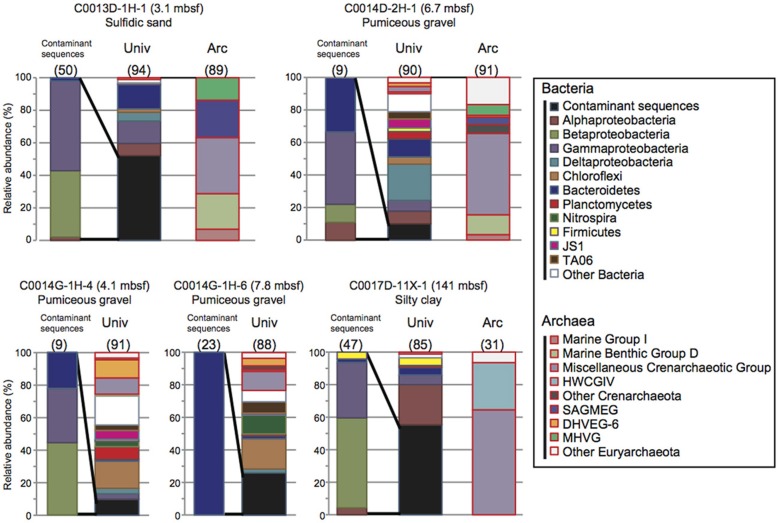
**Microbial community structures based on 16S rRNA gene clone libraries in the core samples obtained during IODP Expedition 331**. The phylotypes that exhibited greater than 97% similarity to the drilling mud fluid sequences were defined as possible contaminants. Univ; bacterial and archaeal phylotype composition determined using the universal primer set. Arc; archaeal phylotype composition determined using the Archaea-specific primer set. The numbers in parentheses indicate the number of clones.

The 16S rRNA gene clone analysis provided microbial community compositions in the subseafloor core samples at Sites C0013, C0014, and C0017. To identify possible indigenous microbial phylotypes, we attempted to discriminate potential contaminants from the drilling fluids in the phylotype compositions by sequence similarity analysis. The 16S rRNA gene phylotype compositions in which the clonal abundance of possible contaminated phylotypes was >10% are presented in Figure 3. Only two samples (C0013D-1H-1, 3.1 mbsf; and C0017D-11X-1, 141 mbsf) seemed to have microbial 16S rRNA gene phylotype compositions that were highly affected by possible contaminants from the drilling fluids (>50% of clonal abundance comprised similar sequences of phylotypes originally detected in the drilling fluids). Most of the potential contaminant sequences were derived from Beta- and Gammaproteobacteria and Bacteroidetes. The extent of the microbial contamination, which was determined from the clonal abundance of possible contaminant phylotypes, was consistent with the extent of contamination estimated by the PFT concentrations and/or microspheres in the core subsamples (Table 1).

The fluorescence microsphere count indicated high contamination from the drilling fluid in C0013D-1H-1, in which 52% of clone sequences were identified as potential microbial contaminants (Figure 3). A large number of microspheres and a relatively high concentration of PFT were also detected in the interior subsample of WRC C0014D-2H-1 (6.7 mbsf), which was mostly composed of volcaniclastic gravels, breccias, and hemipelagic mud. In this sample, ~10% of clone sequences were regarded as potential contaminants (Table 1 and Figure 3). Similarly, the potential microbial contaminants from the drilling fluids shared 10 and 16% of the clone sequences of C0014G-1H-4 and C0014G-1H-6, respectively. Drilling fluid infiltration and disturbance are unavoidable during the drilling and coring operations of very porous, water-absorbent or fragile layers consisting of coarse-grained pumiceous gravel, breccia and hydrothermally altered minerals. These layers in Sites C0013 and C0014 might act as a hydrothermal conduit or impermeable cap-rock overlying a hydrothermal fluid reservoir, which provide a potential habitat for subvent microbial communities. Hence, microbiological and geochemical analyses should be conducted concomitantly with careful contamination assessments.

The 16S rRNA gene phylotypes in the core subsamples with less than 97% similarity to the possible contaminant sequences in the original drilling fluids should be derived from the indigenous microbial communities in the subseafloor environments of the Iheya North hydrothermal system. The detailed phylotype compositions of each core samples, their vertical profiles, and the interpretation of the results with respect to hydrogeological and geochemical interactions will be discussed elsewhere. However, several 16S rRNA gene phylotypes that passed through the initial contamination screening were likely associated with the potential indigenous microbial populations in the hydrothermally active subseafloor habitats. Phylotypes related to thermophilic bacterial taxa such *as Thermotogae and Thermodesulfobacteria* were found in samples C0014D-2H-1, C0014G-1H-4, and C0014G-1H-6. In addition, archaeal phylotypes, which were not detected in any of the drilling fluids, should be derived from the indigenous archaeal populations. In particular, the archaeal phylotypes related to anaerobic methanotrophs (ANME) found in samples C0014D-2H-1 and C0014G-1H-4 have often been observed in hydrothermally affected subseafloor sediments (Teske et al., 2002; Nunoura et al., 2010; Yanagawa et al., 2013). In the deep core sample of Site C0017 (C0017D-11X-1, 141mbsf), archaeal phylotypes affiliated with Hot Water Crenarchaeotic Group IV (HWCGIV; alternatively classified as the Terrestrial Hot Spring Crenarchaeotic Group [THSCG] or UCII) were detected (Figure 3). HWCGIV sequences have also been reported in microbial habitats associated with deep-sea hydrothermal activities (Schrenk et al., 2003; Nunoura et al., 2010; Yoshida-Takashima et al., 2012).

These results suggest that detailed contamination assessments, including the development of a library of potential contaminant phylotypes, are an important component of any investigation of potentially indigenous microbial populations and their functions. Most of the potential contaminant sequences were derived from Beta- and Gammaproteobacteria and Bacteroidetes (Figure 3) A few sequences were closely related (>97% sequence similarity) to previously reported contaminant phylotypes such as *Pseudomonas, Halomonas* and *Vibrio* (Masui et al., 2008; Santelli et al., 2010). The remaining sequences have not been identified as contaminant sequences. Our knowledge of contaminant sequences is still lacking. As described above, large sequence databases based on pyrosequencing results obtained with various drilling/coring systems and drilling fluid components will be needed to discriminate contamination-specific phylotypes from the indigenous microbial community. Furthermore, microbial activity measurements, molecular quantification, isolation of microbial components, and metagenomic and metatranscriptomic analyses will shed light on the indigenous microbial communities and their functions in certain boundary habitats of the subseafloor environments beneath the Iheya North hydrothermal field.

## Conclusions

This study reports the first quantitative contamination tests of riser-less drilling cores using PFTs and fluorescent microspheres during the IODP Expedition on the D/V *Chikyu*. The contamination assessments in the subseafloor hydrothermal environments revealed that the microspheres were not suitable for the high-temperature environments, while the PFT assessment was found to be useful. Microbiological contamination from the drilling mud fluids was also evaluated by molecular biological analysis. The 16S rRNA gene phylotype composition in the drilling mud fluids varied among different sites, holes and depths and differed from previously reported contamination sequences. These results indicate the necessity of periodic sampling of drilling fluids to monitor microbial community structures to distinguish microbial contaminants from potentially indigenous populations in samples. Sequences related to those obtained from the drilling mud were detected from the core interiors. Such contamination likely has a large impact on samples near the potential boundary between the habitable and uninhabitable zones of microbial life, *i.e.*, very low biomass environments. In fact, more than half of the 16S rRNA gene sequences in samples C0013D-1H-1 and C0017D-11X-1 were possible contaminants from the drilling fluids. In the near future, aseptic drilling using filter-sterilized drilling fluid will be required to evaluate indigenous populations thriving in the subseafloor. Alternatively, freshly prepared seawater gel mud could be used as the drilling fluid, as 16S rRNA genes were not PCR-amplified from this material.

### Conflict of interest statement

The authors declare that the research was conducted in the absence of any commercial or financial relationships that could be construed as a potential conflict of interest.
